# Decolonization potential of 0.02% polyhexanide irrigation solution in urethral catheters under practice-like in vitro conditions

**DOI:** 10.1186/s12894-018-0362-3

**Published:** 2018-05-24

**Authors:** Florian H. H. Brill, Henrik Gabriel, Holger Brill, Jan-Hendrik Klock, Joerg Steinmann, Andreas Arndt

**Affiliations:** 1 Dr. Brill + Partner GmbH Institute for Hygiene and Microbiology, Stiegstück 34, 22339 Hamburg, Germany; 2 Institute of Medical Microbiology, University Hospital Essen, Essen, Germany; 3 Institute of Clinical Hygiene, Medical Microbiology and Infectiology, Paracelsus Medical University, Klinikum Nürnberg, Nuremberg, Germany; 4 Department of Research and Development, B. Braun Medical Ltd., Sempach, Switzerland

**Keywords:** Bacterial decolonization, Biofilm, Polyhexanide, Urinary catheter, Urinary tract infection

## Abstract

**Background:**

Long-term use of indwelling urethral catheters is associated with high risk of urinary tract infection (UTI) and blockage, which may in turn cause significant morbidity and reduce the life of the catheter. A 0.02% polyhexanide irrigation solution has been developed for routine mechanical rinsing together with bacterial decolonization of suprapubic and indwelling urethral catheters.

**Methods:**

Using a practice-like in vitro assay and standard silicon catheters, artificially contaminated with clinically relevant bacteria, experiments were carried out to evaluate the bacterial decolonization potential of polyhexanide vs. 1) no intervention (standard approach) and 2) irrigation with a saline (NaCl 0.9%) solution. Swabbing and irrigation was used to extract the bacteria.

**Results:**

Irrigation with polyhexanide reduced the microbial population vs. the control catheters by a factor of 1.64 log_10_ (swab extraction) and by a factor of 2.56 log_10_ (membrane filtration). The difference in mean microbial counts between the two groups (0.90) was statistically significant in favor of polyhexanide when the liquid extraction method was used (*p* = 0.034). The difference between the two groups using the swab extraction method did not reach statistical significance.

**Conclusions:**

The saline and polyhexanide solutions are able to reduce bacterial load of catheters, which shows a combined mechanical and antimicrobial effect. Further research is required to evaluate the long-term tolerability and efficacy of polyhexanide in clinical practice.

## Background

The long-term use of indwelling urethral and suprapubic catheters to manage intractable urinary incontinence and retention is commonplace in both hospital and especially community healthcare settings [1–9]. Surveys of nursing homes across Europe and the US have shown that between 8 to 10% of residents have indwelling urinary catheters [3, 4]. The long-term catheterization patient population is a heterogeneous group, many of which are elderly people who have chronic disabilities [10, 11]. Long-term catheterization can lead to significant patient morbidity and mortality caused by associated complications [10]. The most common complications that occur are urinary tract infection (UTI) and catheter blockage, which can affect up to 70% of catheterized patients [9–11]. Enteric pathogens (e.g. *Escherichia coli*) are most commonly responsible; however, *Pseudomonas* species, *Enterococcus* species, *Staphylococcus aureus*, coagulase-negative staphylococci, *Enterobacter* species and yeasts are also known to cause infection [12]. Bacteria gain access to the urinary tract either extra- or intraluminally and form biofilm colonies, which may adhere to the catheter surface and drainage bag [12–15]. As well as providing a source of infection, biofilm formation is also implicated in encrustation and blockage of catheters [14].

Bacteria within biofilms are morphologically and physiologically different from planktonic bacterial cells and are often resistant to systemic antibiotic treatment, making them difficult to eradicate [13]. However, there is evidence suggesting that physical removal, i.e. mechanical rinsing, is the best method of biofilm elimination, and regular cleansing is required to prevent regrowth of the bacteria [16]. Nevertheless, current guidance does not recommend an active approach for catheter management [17, 18]. The standard clinical approach is not to intervene and therefore removal of the catheter may be the only option [3].

Broad-spectrum antimicrobial agents such as polyhexanide that kill microorganisms have shown to be appropriate agents to use for mechanical rinsing and removal of biofilm across a range of applications [16, 19]. The aim of this study was to investigate whether taking an active approach with a mechanical irrigation with normal saline solution and a combined approach with a polyhexanide solution is efficient for decolonizing urethral catheters. The bactericidal activity of the polyhexanide solution was tested in vitro using a suspension assay. In addition, the bacterial decolonization potential was tested using a practice-like in vitro assay with standard catheters, which were artificially contaminated. The polyhexanide solution and irrigation with a normal saline solution were compared to no intervention (standard approach).

## Methods

The decolonization activity of a polyhexanide solution (Uro-Tainer® 0.02% Polyhexanide, B. Braun Medical Ltd., Sempach, Switzerland) and normal saline solution was assessed in the presented study. For the time being polyhexanide is used in wound antiseptics with 0.02% and 0.04% concentration [20]. The 0.02% concentration is recommended for clean wounds. As in the application for transurethral catheter decolonization in most cases clean conditions without blood are expected, the lower concentration has been selected to reduce the risk for irritation in patients.

### Bactericidal activity in suspension assay

The suspension tests were performed according to EN 13727 [21] against the following six bacteria strains: *Staphylococcus aureus (*ATCC 6538*)*, *Enterococcus hirae (*ATCC 10541*)*, *Escherichia coli K12 (*ATCC 11229, *Proteus mirabilis (*ATCC 14153*)*, *Pseudomonas aeruginosa (*ATCC 15442*)*, *Klebsiella pneumoniae (*ATCC 16609*)*. The bacteria were incubated for 48 h at 36 ± 1 °C. They were exposed to the polyhexanide solution at 50 and 80% *v*/v for 5, 15, 30 and 60 min under clean conditions (0.3 g/L bovine serum albumin). A combination of 80 g/L polysorbate 80, 60 g/L saponin, 6 g/L lecithin, 20 g/L sodium dodecyl sulphate in A. dest. was validated and used as neutralizing solution.

### Catheter decolonization assay

The test bacteria were *Escherichia coli* (ATCC 11229), *Proteus mirabilis* (ATCC 14153) and methicillin-resistant *Staphylococcus aureus* (MRSA) (ATCC 33592). The devices used in all experiments were 41 cm long transurethral silicon balloon catheters with a nelaton-top, size ch. 18 (B. Braun Melsungen Ltd., Germany).

**Fig. 1:**
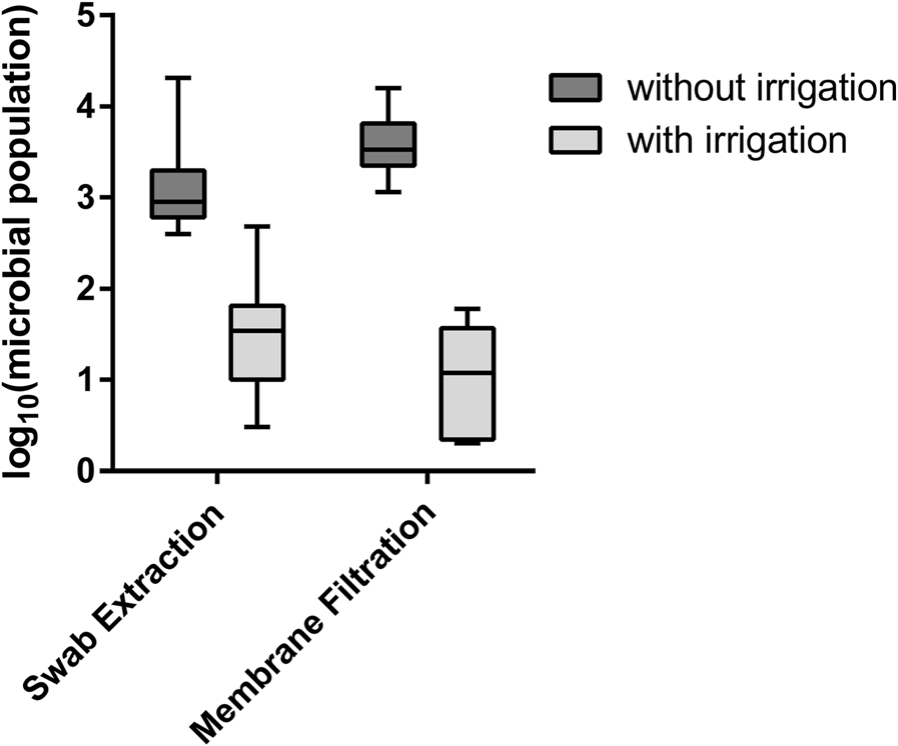
Catheter decolonization results – Polyhexanide (with irrigation) versus without irrigation. The Difference between with and without irrigation groups was statistically significant (*p* = 0.012)

The experimental set up of the in-vitro decolonization assay was as follows:

- The catheters were contaminated with 5 ml of a mixed suspension of the test organisms (10^8^ to 10^9^ colony forming units [cfu] per ml simulating worst-case conditions) in caseinpepton-soy bean pepton-bouillon daily for 3 days (Fig. 1).
- The contaminated catheters were incubated for a total of 72 h at 36 °C ± 1 °C.
- All catheters were irrigated twice daily with a flow of 400 ml of the synthetic urine (composition according to EN 1616: 25 g/L urea, 9 g/L NaCl, 2.5 g/L Potassium hydrogenorthophosphate, dipotassium hydrogenorthophosphate, 3 g/L ammonium chloride 2 g/L kreatinin, 3 g/L sodium sulphite in A. dest. + 3 g/L % bovine serum albumin [22]) during the incubation period.
- After incubation and the treatment of the catheter with normal saline or polihexanide or no treatment respectively the catheters were irrigated with 100 ml of neutralizer solution (1 g/L polysorbate 80, 1 g/L lecithine, 1 g/L histidine, 2 g/L Sodium dodecyl sulphate) and the microbial count in the solution was determined by membrane filtration of 50 ml and with serial dilution tests (liquid extraction method).
- In addition, all catheters were cut with a sterile scalpel and the content of the inner lumen of the catheters was extracted with a sterile cotton swab. The swab was suspended in normal saline solution and the microbial count was determined with a serial dilution assay (swab extraction method).
- The endpoint, i.e. log_10_ reduction factor (RF) in microbial count, was calculated for the treatment groups vs. the control no treatment group.
- The statistical difference between the groups was evaluated by the one-sided unpaired Wilcoxon rank sum test [23].

In the first experiment according to above described methodology a total of 20 contaminated catheters were included. Ten were used as growth control in the no treatment group and 10 were treated with polyhexanide solution by connecting the device to the catheter as described in the instructions for use. After the exposure time of 5 min the microbial count was determined.

The second catheter decolonization experiment was similar to the first experiment. A total of 30 catheters were included. Ten catheters were treated with 100 ml 0.9% NaCl solution with an exposure time of 5 min. Additional 10 catheter were treated with 100 ml of the polihexanide solution with an exposure time of 5 min. The remaining 10 catheters were used as growth control and not treated.

## Results

In the suspension test assay according to EN 13727 the polyhexanide solution was shown to have bactericidal activity in vitro against *Staphylococcus aureus, Enterococcus hirae, Pseudomonas aeruginosa, Escherichia coli, Proteus mirabilis* and *Klebsiella pneumonia* under clean conditions (Table 1).

**Table 1:** Bactericidal activity of polyhexanide 0.02% solution against different reference strains

Test Sample/ Test Organism Microbial Count Control NO	Ig-reduction factor after minutes				
	Conc. in %	5	15	30	60
*Staphylococcus aureus* (ATCC 6538), NO = 7.62	50.00	≥ 7.62	≥ 7.62	≥ 7.62	≥ 7.62
	80.00	≥ 7.62	≥ 7.62	≥ 7.62	≥ 7.62
*Enterococcus hirae* (ATCC 10541), NO = 7.42	50.00	≥ 7.42	≥ 7.42	≥ 7.42	≥ 7.42
	80.00	≥ 7.42	≥ 7.42	≥ 7.42	≥ 7.42
*Escherichia coli* K12 (ATCC 11229), NO = 7.26	50.00	n.c.	≥ 6.56	≥ 7.26	≥ 7.26
	80.00	3.93	≥ 7.26	≥ 7.26	≥ 7.26
*Proteus mirabilis* (ATCC 14153), NO = 7.76	50.00	≥ 7.76	≥ 7.76	≥ 7.76	≥ 7.76
	80.00	≥ 7.76	≥ 7.76	≥ 7.76	≥ 7.76
*Pseudomonas aeruginosa* (ATCC 15442), NO = 7.35	50.00	n.c.	5.58	≥ 7.43	≥ 7.43
	80.00	≥ 7.35	≥ 7.43	≥ 7.43	≥ 7.43
*Klebsiella pneumonia* (ATCC 16609), NO = 7.66	50.00	5.18	≥ 7.66	≥ 7.66	6.96
	80.00	≥ 7.66	≥ 7.66	≥ 7.66	6.96

*n.c.* not calculable

The results of the first catheter decolonization assay are summarized in Fig. 1. The control catheters (standard approach i.e. no intervention) had a mean microbial population of 3.12 log_10_ (range 2.60–4.31 log_10_) when measured using the swab extraction method and 3.57 log_10_ (range 3.06–4.20 log_10_) when the membrane filtration method was used. The catheters irrigated with the polyhexanide solution had a mean microbial population of 1.47 log_10_ (range 0.48–2.68 log_10_) when measured using the swab extraction method and 1.01 log_10_ (0.30–1.60 log_10_) when the membrane filtration method was used. The difference in the mean microbial count between the two groups was statistically significant in favor of the polyhexanide solution for both methods of analysis (*p* = 0.012).

Irrigation of the catheters with the polyhexanide solution reduced the microbial population compared to that of the control catheters by a factor of 1.64 log_10_ (1.12–2.30 log_10_) when measured using the swab extraction method and by a factor of 2.56 log_10_ (1.46–3.20 log_10_) using the membrane filtration method.

The results of the second catheter decolonization assay are summarized in Table 2. The catheters irrigated with NaCl 0.9% had a mean microbial population of 2.37 log_10_ (± 0.196) when measured using the swab extraction method and 1.70 log_10_ (± 0.458) when the membrane filtration method was used. The catheters irrigated with the polyhexanide solution had a mean microbial population of 2.06 log_10_ (± 0.514) when measured using the swab extraction method and 0.80 log_10_ (± 0.748) when the membrane filtration method was used. The difference in mean microbial count between the two groups (0.90) was statistically significant in favor of the polyhexanide solution when the liquid extraction method was used (*p* = 0.034). However, the difference between the two groups using the swab extraction method was not statistically significant (*p* = 0.173).

**Table 2:** Catheter decolonization results – polyhexanide 0.02% versus NaCl 0.9%

Catheter	Swab Extraction Method			Membrane Filtration Method		
	log with NaCl	log with polyhexanide	Difference NaCl –polyhexanide	log with NaCl	log with polyhexanide	Difference NaCl –polyhexanide
1	2.34	2.48	−0.13	1.00	1.00	0.00
2	2.48	1.48	1.00	1.00	1.00	0.00
3	2.48	1.30	1.18	2.00	0.00	2.00
4	2.48	1.48	1.00	2.00	0.00	2.00
5	2.48	1.48	1.00	2.00	2.00	0.00
6	2.48	2.48	0.00	2.00	2.00	0.00
7	2.48	2.48	0.00	1.00	1.00	0.00
8	2.48	2.48	0.00	2.00	1.00	1.00
9	2.08	2.48	−0.40	2.00	0.00	2.00
10	1.90	2.48	−0.57	2.00	0.00	2.00
Mean	2.37	2.06	0.31^a^	1.70	0.80	0.90^b^
Standard deviation	0.196	0.514	0.628	0.458	0.748	0.943
No of catheters	10			10		

^a^The difference between the polyhexanide and NaCl groups is not statistically significant (*p* = 0.173) for the swab extraction method

^b^The difference between the polyhexanide and NaCl groups is statistically significant (*p* = 0.034) for the liquid extraction method

## Discussion

This study has demonstrated that the polyhexanide solution has bactericidal activity in vitro against microorganisms which have been commonly associated with development of UTI in catheterized patients [12, 15]. Furthermore, this is the first in vitro study to demonstrate the potential role of polyhexanide for bacterial decolonization of urinary catheters.

The current literature on bacterial decolonization of urethral catheters is largely restricted to treatment with a range of systemic antibiotic regimens [24]. In a study by Jones et al. it has been shown that MRSA was able to colonize a silastic rubber surface even in the presence of prophylactic vancomycin or rifampicin [25]. Furthermore, other studies have shown that the lowest concentration required to eradicate bacterial biofilm for many antibiotics may exceed the maximum therapeutic dose level [26–29].

As an alternative to systemic antibiotic treatment efforts have been made to develop catheters coated with hydrophilic gels or antibacterial agents (such as silver) to prevent bacterial adhesion [30] .However, none of these developments have been shown to resist biofilm formation, especially in patients with *Proteus mirabilis* UTI [30]. One explanation for this lack of success is that this approach does not involve mechanical rinsing or irrigation.

In comparison to antibiotics and use of coated catheters it is suggested that polyhexanide might provide an effective, non-systemic approach to bacterial decolonization of urinary catheters. Polyhexanide has been successfully used for bacterial decolonization and prevention of biofilm formation in wound management [19, 31–35]. In an in vitro study rinsing with polyhexanide solution significantly reduced MRSA biofilm at 48 and 72 h compared to two saline solutions (*p* < 0.05) [32]. In addition to its bactericidal properties, polyhexanide has also been shown to have anti-adhesive properties due to its chemical nature (i.e. cationic), which may have the potential to help prevent biofilm formation [31].

The present study has shown that mechanical rinsing with 0.9% NaCl solution and a 0.02% solution of polyhexanide was shown to be significantly and consistently more effective at reducing the bacterial colonization of the catheters compared to no intervention (standard approach). However, there was no significant difference seen in microbial count between the polihexanide compared to the control groups when the swab extraction method was used.

The results of this study were achieved in experiments that were designed to replicate practice-like conditions as closely as possible. Silicone catheters used routinely in practice were contaminated with a combination of clinically relevant bacteria (e.g. *Proteus mirabilis* and MRSA) and a solution of synthetic urine and incubated for several days at body temperature. The polyhexanide solution and the saline solution were used according to the manufacturer’s instructions to rinse the catheters for 5 min as used in clinical practice. However, the study has some limitations: the experiments were performed with only one type of catheter. The inoculation with mixed bacterial strains was not standardized in terms of growth of the included species. The microbial counts of the three test bacteria were not determined separately. Therefore, it is possible that the effect of the treatments is not balanced against all bacteria. Also it is possible that some bacteria turned into viable, but not culturable (VBNC) status especially after antimicrobial treatment. As we used standard test bacteria under laboratory conditions on solid media, it is unlikely but possible that this has influenced the results. In addition, practice-like in vitro conditions cannot replace in vivo studies and therefore the results may not directly translate into the clinical setting. Further research is required to demonstrate the efficacy of the polyhexanide as well as 0.9% NaCl solution across a range of catheter types and materials available in clinical practice. Research is also required in patients to evaluate the tolerability and clinical effectiveness of the polyhexanide solution when used for routine catheter maintenance in the short- and long-term.

In summary, the results of this study have shown that a 0.02% polihexanide and 0.9% NaCl solution is able to significantly reduce bacterial load of catheters, which shows a combined mechanical and antimicrobial effect. Further research is required to demonstrate the tolerability and efficacy of the polyhexanide solution in daily clinical practice.

## Abbreviations

MRSA: Methicillin-resistant *Staphylococcus aureus*
RF: Reduction factor
UTI: Urinary tract infection
